# Influence of organic rice production mode on weed composition in the soil seed bank of paddy fields

**DOI:** 10.3389/fpls.2022.1056975

**Published:** 2022-11-21

**Authors:** Pinglei Gao, Haoyu Wang, Shiwen Deng, Erjia Dong, Qigen Dai

**Affiliations:** ^1^ Jiangsu Key Laboratory of Crop Genetics and Physiology, Jiangsu Co-Innovation Center for Modern Production Technology of Grain, Research Institute of Rice Industrial Engineering Technology, Yangzhou University, Yangzhou, China; ^2^ Jiangsu Key Laboratory of Crop Cultivation and Physiology, Jiangsu Co-Innovation Center for Modern Production Technology of Grain, Research Institute of Rice Industrial Engineering Technology, Yangzhou University, Yangzhou, China; ^3^ Anhui Pujiwei Modern Agriculture Group Co., Ltd., Tongling, China

**Keywords:** organic agriculture, rice-green manure rotation, rice integrated planting–breeding, weed composition, weed biodiversity

## Abstract

**Introduction:**

It is of great significance to determine the composition of the soil weed seed bank under different organic rice production modes to provide decision making support for rational integrated weed management in organic rice production.

**Methods:**

The soil weed seed bank of the four dominant organic production modes, namely, rice-green manure rotation (RG), rice monoculture (RM), rice-crayfish coculture (RC) and rice-duck coculture (RD), with different numbers of consecutive planting years (3 to 10 years) in different sites in Jiangsu Province were investigated to determine the influence of organic rice production mode on weed composition.

**Results and Discussion:**

There were significant differences in the weed composition in the soil seed bank among the four organic rice production modes. The most dominant weed group was broadleaf weeds in the soil seed bank under the RG and RM modes; however, under the RM mode, the most dominant weed species were sedge and grass weeds. Sedge and grass weeds dominated the soil seed bank of the RC and RD modes, respectively. Therefore, specific weed management strategies could be formulated based on the differences in weed composition under different organic rice production modes. The application of organic fertilizer and irrigation were identified as primary factors associated with the differences in weed composition in the soil seed banks, which had higher effects on the weed composition than hand weeding. Consequently, fertilization and irrigation strategies that alter weed composition could be used as improved weed management program components in organic rice production systems. Long-term organic rice planting is beneficial for increasing weed diversity in paddy fields. Our results indicated that weed species diversity increased and weed community evenness and dominance decreased with the increase in the number of consecutive planting years under all four organic rice production modes.

## Introduction

Rice (*Oryza sativa*) is one of China’s major food crops, where it accounts for approximately 18% of the world’s rice planting area, and is a staple food resource for more than half of the global population (Kraehmer et al., 2016; Arunrat et al., 2021). Conventional rice production is heavily dependent on synthetic chemical fertilizers and pesticides to ensure rice yield. However, long-term heavy application of synthetic chemical fertilizers and pesticides has contributed to environmental hazards around the world, including water contamination, soil erosion, biodiversity loss and pesticide resistance, and is also accompanied by an increase in rice production costs (Poudel et al., 2002; Brown and Funk, 2008; Shaner, 2014). Few would disagree that it is necessary to develop and promote ecologically and environmentally sustainable agricultural systems to cope with the environmental crisis caused by conventional agriculture. Organic agriculture is considered a promising solution for reducing the negative impacts of conventional agriculture on the environment due to the lower use of off-farm inputs, higher input‒output efficiency and environmental benefits (Ikemura et al., 2008; Seufert et al., 2012; Arunrat et al., 2021). Along with increasing public knowledge about health and healthy lifestyles, the demand for organic products by consumers all over the world is increasing (Hazra et al., 2018). Organic agriculture is defined as a food production system that relies on ecological processes, biodiversity and cycles adapted to local conditions to maintain soil, ecosystem and human health (Suh, 2015). Based on the ecological characteristics of paddy fields and to meet the fertilizer and pest control demands during rice planting, in addition to basic rice monoculture, a variety of organic rice production modes have been developed. These include rice-green manure rotation, which uses green manure to reduce the application of organic fertilizer (Hazra et al., 2018; Du et al., 2022), and rice integrated planting–breeding, which makes use of the aquatic environment of paddy fields to achieve mutual benefits for rice planting and waterfowl or aquatic animal breeding (Nair et al., 2014; Pirdashti et al., 2015; Xu et al., 2019).

Weed competition is one of the major factors constraining rice yield, especially in organic rice production, in which chemical herbicides have been prohibited (Hokazono et al., 2009). Weed infestation can usually reduce rice yield by 10–20%, and when the field is severely infested, the yield can be reduced by more than 50% or even to no harvest (Rathod and Somasundaram, 2017). The existence of weed seeds in the soil seed bank is the main source leading to the continuous infestation of weeds in farmland (Barberi and Lo Cascio, 2001). The composition and succession of the weed community are closely related to the soil weed seed bank, which is influenced by farming practices, such as tillage and fertilization practices, rotation and continuous cropping and weeding methods (Yin et al., 2005; Chamanabad et al., 2009; Chauhan and Opena, 2012; Santínmontanyá et al., 2013). Studies on the composition of weed seed banks and the factors affecting their formation in farmland soils could help predict the infestation of weed populations and improve decision-making for managing specific weed problems (Chauhan and Johnson, 2010; Gao et al., 2020).

Rice is the first and foremost grain crop in Jiangsu Province, and its perennial planting area and yield account for approximately 42% and 55% of the total planting area and yield of grain crops in the province, respectively (National Bureau of Statistics, 2020). With the improvement in people’s living standards and environmental awareness, the demand for organic rice has increased, and a variety of organic production modes have been developed, such as rice monoculture, rice-green manure rotation, rice-duck coculture, rice-crab coculture, rice-crayfish coculture and rice-frog coculture in Jiangsu Province. According to Du et al. (2022), the total planting area of organic rice in Jiangsu Province was 4410 ha. Among the organic production modes, rice-green manure rotation (RG), rice-duck coculture (RD), rice-crayfish coculture (RC) and rice monoculture (RM) were the four dominant organic rice production modes, and weed control in organic rice fields mainly depended on hand weeding. However, hand weeding is becoming problematic due to the shortage of labor and increasing labor costs in agricultural production (Liu and Zhou, 2022). Therefore, it is of great significance to determine the composition characteristics of the soil weed seed bank under different organic rice production modes to provide decision-making support for rational integrated weed management in organic rice production. In the present study, the soil weed seed bank under the four dominant organic production modes (RG, RM RC and RD) with different numbers of consecutive planting years (3 to 10 years) in different sites in Jiangsu Province were investigated to: (i) determine whether the composition of the weed seed bank is similar among different sites under the same organic rice production mode; (ii) identify the main farming practices leading to the differences in the composition of weed seed banks under different organic rice production modes; and (iii) establish the variation in the total seed density and diversity of the weed seed bank over consecutive years under a certain organic production mode.

## Materials and methods

### Study site

**Figure 1:**
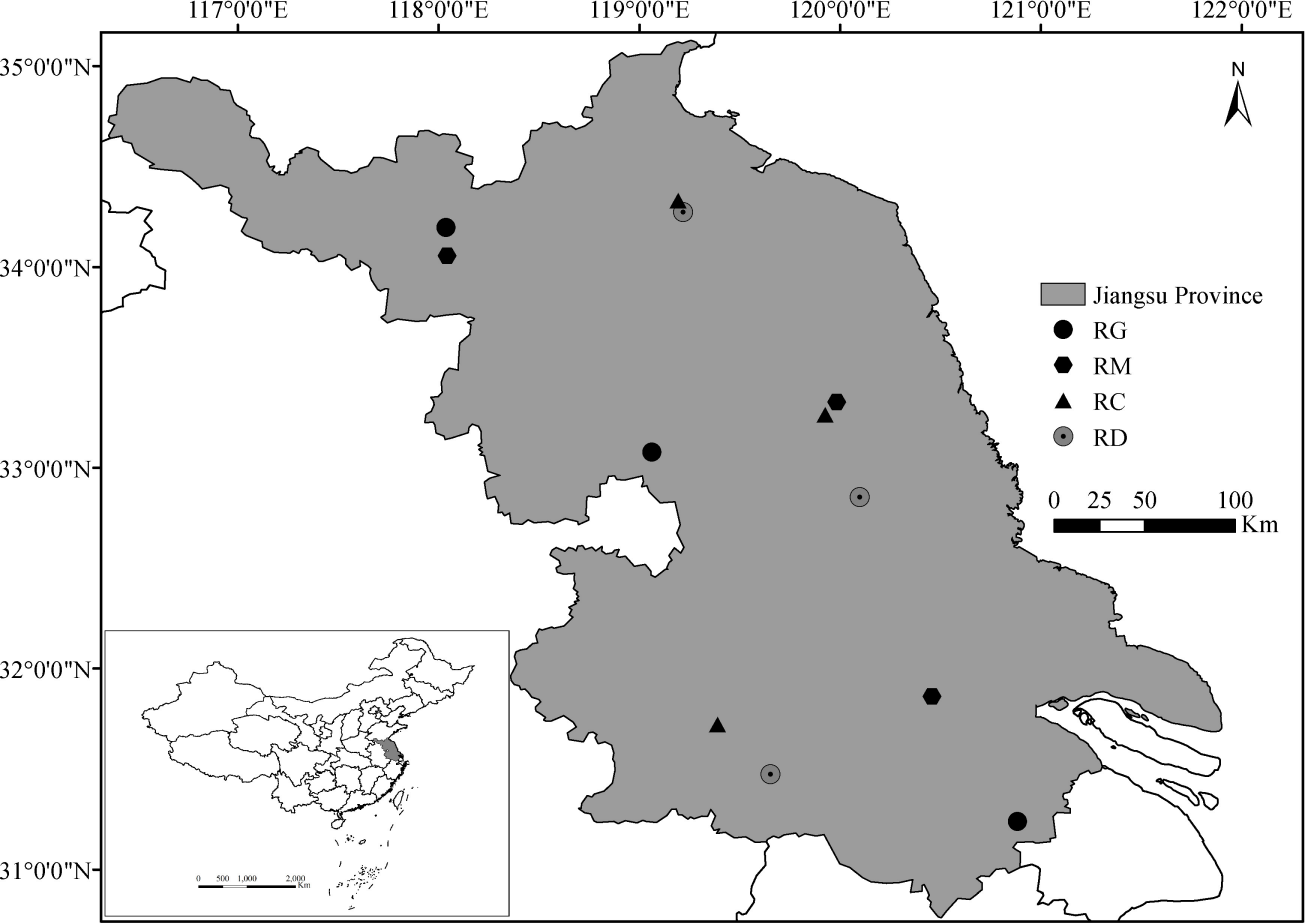
Sampling sites of the four organic rice production modes in Jiangsu Province. RG, rice-green manure rotation; RM, rice monoculture; RC, rice-crayfish co-culture; RD, rice-duck co-culture.

Jiangsu Province is located in the center of the east coast of mainland China (30°45′ to 35°20′N, 116°18′ to 121°57′E, **Figure 1**). The land area of Jiangsu is 103229.17 km^2^, of which the plain area accounts for 86.89%. There are many rivers and lakes in Jiangsu, so it has a dense crisscross water network. Jiangsu belongs to the East Asian monsoon climate zone, with an annual average temperature between 13.6°C and 16.1°C and precipitation between 704 mm and 1250 mm; affected by the monsoon, the temperature and precipitation in northern and southern Jiangsu are significantly different (www.weather.com.cn). The unique landform, hydrological and climatic environment of Jiangsu make it the most suitable territory for rice planting, and various organic rice production modes are widely distributed throughout the province.

### Data collection

In the north, middle and south of Jiangsu Province, one sampling site for organic rice fields under the RG, RM, RC and RD modes was selected, respectively, for a total of 12 sampling sites (**Figure 1**), which were sampled in early November after each rice harvest in 2020. For weed seed bank sampling, three nonadjacent fields (area of 0.2–0.4 ha per field) were selected at each sampling site, in which nine 1 m^2^ (1 m × 1 m) quadrats were positioned in accordance with the inverted “W” 9-point sampling method (Thomas, 1985), and eighteen soil cores (3.5 cm in diameter and 15 cm deep, which is equal to the plow layer) were equidistantly sampled from each quadrat. After being smashed and air-dried, soil samples from each sampling site were passed through a 4-mm sieve to remove large stones and debris. Subsequently, each soil sample was divided into five parts, and the seed content of each part was equated to a seed density of 0.016 m^2^ in 15 cm deep soil, three of which were determined by elutriation (Wiles et al., 1996). After elutriation, the remnant of each divided part was air-dried and examined under a binocular dissecting mirror (maximum magnification of 400X) to determine the weed species and number of each species.

In addition, the location, number of consecutive planting years, and farming practices, including rice varieties, tillage, irrigation, organic fertilizer application, green manure (Chinese Milk Vetch (*Astragalus sinicus*)) burying, juvenile crayfish and duckling input, and weeding were also recorded during sampling. Two rice varieties, Nanjing9108 and Nanjing46, was continuously planted in each sampling site of different organic rice planting modes, respectively. Rice seedlings were transplanted by transplanting machine from June 15 to 21 of every year at a hill spacing of 12 cm by 30 cm (27.75 by 104 hills·ha^-1^) with three seedlings per hill in every sampling field. The irrigation of the RG and RM mode was the same as that of conventional rice production, while the amounts of irrigation were higher in the RC and RD mode to provide suitable living environments for crayfish and duck. Before transplanting, all organic rice production modes conducted the same three times of tillage, including two rotary tillage (15 cm depth before and 12 cm depth after irrigation) and one harrow tillage (5 cm depth), besides, an additional rotary tillage (15 cm depth) was carried out in the RG mode to bury green manure 30 days before rice transplanting. The organic fertilizer was commercial organic fertilizer which consisted of 45.4% organic matter, 2.0% N, 2.9% P_2_O_5_, 1.2% K_2_O, and 29.1% water and applied before tillage in all sampling fields. The input of juvenile crayfish and duckling input was 2 weeks after transplanting every year in the RC and RD mode. Weeds were uprooted and removed two times at middle tillering stage (around 20 days after transplanting) and 3 weeks later every year in each sampling field by hand weeding. The details of the location, number of consecutive planting years and farming practices, including rice varieties, times of tillage per year, annual irrigation volume, annual application amount of organic fertilizer, fresh weight of green manure buried every year, annual input of juvenile crayfish and duckling, and annual hand weeding time in each sampling site were presented in **Table 1**.

**Table 1:** The consecutive plant years and farming practices of each organic rice production mode in each sampling site.

Location^a^	PM^b^	CP^c^	RV^d^	T^e^	I^f^	OF^g^	GM^h^	CF^i^	D^j^	HW^k^
TS	RG	3	Nanjing9108	4	5.6	6.0	27.6	0	0	41
QF	RG	10	Nanjing9108	4	5.4	5.9	29.6	0	0	43
HS	RG	5	Nanjing46	4	5.6	6.1	26.4	0	0	42
WJ	RM	3	Nanjing9108	3	5.6	13.1	0	0	0	41
HJ	RM	8	Nanjing9108	3	5.5	12.8	0	0	0	43.5
TL	RM	5	Nanjing46	3	5.6	12.9	0	0	0	43
YS	RC	6	Nanjing9108	3	7.8	5.4	0	442.5	0	19.5
QN	RC	3	Nanjing9108	3	8.0	5.5	0	427.5	0	21.5
XB	RC	8	Nanjing9108	3	7.7	5.3	0	480	0	21
BP	RD	4	Nanjing9108	3	6.5	6.1	0	0	270	24
DY	RD	6	Nanjing46	3	6.3	6.1	0	0	240	23
YX	RD	10	Nanjing46	3	6.1	5.9	0	0	255	21

^a^TS, Tushan Town; Xuzhou City; QF, Qianfeng Town, Huaian City; HS, Huashi Town, Wuxi City; WJ, Weiji Town, Xuzhou City; HJ, Hengji Town, Yancheng City; TL, Tongli Town, Suzhou City; YS, Yishan Town, Liangyungang City; QN, Qinnan Town, Yancheng City; XB, Xuebu Town, Changzhou City; BP, Banpu Town, Liangyungang City; DY, Diaoyu Town, Huaian City; YX, Yangxiang Town, Wuxi City.

^b^Production mode, RG, rice-green manure rotation; RM, rice monoculture; RC, rice-crayfish co-culture; RD, rice-duck co-culture.

^c^Consecutive planting years.

^d^Rice varieties.

^e^Times of tillage per year.

^f^Annual irrigation volume (10^3^m^3^·ha^-1^).

^g^Annual application amount of organic fertilizer (10^3^kg·ha^-1^).

^h^Fresh weight of green manure buried every year (10^3^kg·ha^-1^).

^i^Annual hand weeding time (hours·ha^-1^).

^j^Annual input of juvenile crayfish (kg·ha^-1^).

^k^Annual input of duckling (duckling·ha^-1^).

### Data processing

The soil weed seed bank data were expressed as the number of seeds per square meter. Phytosociological structure was assessed by common parameters, including absolute and relative frequency, density and abundance and importance value (*IV*) for each weed species, which were calculated with the following equations (Lal et al., 2016):


$$Absolute\;frequency\;(AF)\;=\;\frac{number\;of\;quadrats\;with\;species\;present}{total\;number\;of\;quadrats}$$

$$Relative\;frequency\;(RF)\;=\;\frac{absolute\;frequency\;of\;a\;species}{sum\;of\;all\;absolute\;frequencies}$$

$$Absolute\;density\;(AD)\;=\;\frac{total\;number\;of\;individuals\;of\;a\;species}{total\;sampled\;area}$$

$$Relative\;density\;(RD)\;=\;\frac{absolute\;density\;of\;a\;species}{sum\;of\;all\;absolute\;densities}$$

$$Absolute\;abundance\;(AA)\;=\;\frac{total\;number\;of\;individuals\;of\;a\;species}{total\;number\;of\;quadrats\;containing\;that\;species}$$

$$Relative\;abundance\;(RA)\;=\;\frac{absolute\;abundance\;of\;a\;species\;}{sum\;of\;all\;absolute\;abundances}$$

Importance value (*IV*) = *RF* + *RD* + *RA*

The species diversity of the soil weed seed bank was assessed with the following indices (Gao et al., 2020):


$$Shannon\;index\;(H')\;=\;-\sum_{i=1}^{S}\frac{n_{i}}{N}ln\left(\frac{n_{i}}{N}\right)$$

$$Simpson\;index\;(\lambda )\;=\;1-{\sum_{i=1}^{S}\left(\frac{n_{i}}{N}\right)}^{2}$$

$$Evenness\;index\;(E)\;=\;\frac{\lambda^{-1}\;-1}{e^{H'}\;-1}$$

$$Ecological\;dominance\;(C)\;=\;\sum_{i=1}^{S}\frac{n_{i}(n_{i}-1)}{N(N-1)}$$

where *S* is the total number of species in the community, *n_i_* is the number of individuals in species *i*, and *N* is the total number of seeds of all species in a quadrat. *H*´and *λ* are commonly used indices to determine species richness in which *H*´ is more sensitive to dense species, while *λ* is more sensitive to sparse species (Magurran, 1988). *E* reflects the evenness of individual distribution among species, that is, the evenness of species abundance (Lal et al., 2016). *C* refers to the degree to which community dominance is concentrated in one or several species (Gao et al., 2020).

To visualize the differences in weed community composition of soil seed bank among the different organic rice production modes, based on *IV*, nonmetric multidimensional scaling (NMDS) was performed using the metaMDS function in the vegan package in R v.3.6.0 (R Development Core Team, 2019) with 250 ordination runs (Oksanen et al., 2019). Permutational multivariate analysis of variance (PERMANOVA) was also performed to evaluate whether weed communities of soil seed bank differed according to organic rice production mode or sampling site, based on 999 permutations *via* the adonis function in the vegan package in R v.3.6.0. To quantify the effect of farming practices, including rice varieties, times of tillage per year, annual irrigation volume, annual application amount of organic fertilizer, fresh weight of green manure buried every year, annual input of juvenile crayfish and duckling, and annual hand weeding time in each sampling site on the weed community composition, aggregated boosted tree (ABT) analysis was performed using the gbmplus package with 500 trees for boosting (De'ath, 2007) in R v.3.6.0. One-way analysis of variance (ANOVA) was conducted to determine the effects of number of consecutive planting years on the total seed density and species diversity of the soil weed seed bank under different organic rice production modes at a significance level of 5%. The total seed density data and *E* values were log(x+1)-transformed to ensure that they satisfied the assumptions of normal distribution of residuals (Shapiro‒Wilk W-test) and homoscedasticity (Levene’s test). Least significant difference (LSD) tests were also conducted to compare the means of total seed density and species diversity indices at a significance level of 5%. These analyses were conducted using SPSS 20 software (SPSS Inc., Chicago, IL, USA), and figures were generated using OriginPro 2021 (OriginLab, Hampton, MA, USA).

## Results

### Weed community composition in different organic production modes

In total, 61 weed species belonging to 48 genera and 21 families were recorded in the soil seed banks of the four organic rice production modes (**Table 2**). Among the 61 weed species, there were 32 rice-associated weed species. Fourteen species, *Echinochloa crusgalli*, *Leptochloa chinensis*, *Cyperus difformis*, *Cyperus iria*, *Najas minor*, *Ludwigia prostrata*, *Ammannia baccifera*, *Rotala indica*, *Lindernia procumbens*, *Monochoria vaginalis*, *Beckmannia syzigachne*, *Myosoton aquaticum* and *Mazus japonicus*, were widely distributed in all sampled fields. *Cnidium monnieri* and *Glycine soja* were found only in one field under RG. *Ajuga ciliate* only occurred in one field under RM*. Juncellus serotinus* was found only in one field under RC, and *Kyllinga brevifolia* only occurred in one field under RD.

**Table 2:** Weed species of the soil seed banks in different organic rice production modes^a^.

Weed species	RG	RM	RC	RD	Weed species	RG	RM	RC	RD
*Alopecurus japonicus* ^b^	+++	++	++	++	*Aeschynomene indica* ^d^	+	–	+	–
*Beckmannia syzigachne* ^b^	+++	+++	+++	+++	*Carpesium abrotanoides* ^d^	–	++	–	–
*Cynodon dactylon* ^b^	–	++	+	–	*Eclipta prostrata* ^d^	++	++	+	–
*Digitaria sanguinalis* ^b^	+	+++	+++	+	*Lapsana apogonoides* ^d^	+++	+++	+	+
*Eleusine indica* ^b^	–	+++	+	++	*Hemistepta lyrate* ^d^	+	+++	+++	+++
*Leptochloa chinensis* ^b^	+++	+++	+++	+++	*Chenopodium album* ^d^	+	+++	+++	+++
*Sclerochloa dura* ^b^	+	+	–	–	*Polygonum aviculare* ^d^	++	+++	+++	–
*Alopecurus aequalis* ^b^	+++	++	+++	+++	*Polygonum lapathifolium* ^d^	++	+++	++	++
*Echinochloa crusgalli* ^b^	+++	+++	+++	+++	*Polygonum nepalense* ^d^	–	+	+	–
*Panicum bisulcatum* ^b^	–	++	+	+	*Polygonum posumbu* ^d^	–	+++	–	–
*Polypogon fugax* ^b^	+	+++	+++	++	*Polygonum viscosum* ^d^	++	++	–	–
*Setaria viridis* ^b^	+	+++	+	+	*Rumex dentatus* ^d^	–	+++	++	–
*Cyperus difformis* ^c^	+++	+++	+++	+++	*Ludwigia prostrata* ^d^	+++	+++	+++	+++
*Cyperus iria* ^c^	+++	+++	+++	+++	*Geranium carolinianum* ^d^	+	–	+	–
*Fimbristylis miliacea* ^c^	+	++	+	–	*Clematis hexapetala* ^d^	+++	+++	+++	++
*Heleocharis yokoscensis* ^c^	–	+++	+++	+++	*Ammannia baccifera* ^d^	+++	+++	+++	+++
*Juncellus serotinus^c^*	–	–	+	–	*Ammannia multiflora* ^d^	+++	++	++	+++
*Kyllinga brevifolia* ^c^	–	–	–	+	*Rotala indica* ^d^	+++	+++	+++	+++
*Scirpus juncoides* ^c^	++	++	+++	++	*Galium aparine* ^d^	+	+	++	+++
*Scirpus triangulatus* ^c^	+++	++	+++	+++	*Cnidium monnieri* ^d^	+	–	–	–
*Scirpus wallichii* ^c^	–	+	+++	+++	*Capsella bursa-pastoris* ^d^	+++	++	–	++
*Scirpus yagara* ^c^	+	+	–	++	*Rorippa indica* ^d^	+	+	++	+
*Ajuga ciliate* ^d^	–	+	–	–	*Myosoton aquaticum* ^d^	+++	+++	+++	+++
*Najas foveolate* ^d^	–	++	–	–	*Lindernia procumbens* ^d^	+++	+++	+++	+++
*Najas minor* ^d^	+++	+++	+++	+++	*Mazus japonicus* ^d^	+++	+++	+++	+++
*Acalypha australis* ^d^	+	–	–	+	*Veronica peregrina* ^d^	+	+	–	+++
*Astragalus sinicus* ^d^	++	–	–	–	*Veronica undulata* ^d^	–	+	–	++
*Glycine soja* ^d^	+	–	–	–	*Monochoria vaginalis* ^d^	+++	+++	+++	+++
*Medicago polymorpha* ^d^	+	–	+	–	*Alisma plantago-aquatica* ^d^	–	+	+	++
*Vicia gigantea* ^d^	+	–	+	–	*Trigonotis peduncularis* ^d^	–	–	+	+
*Vicia hirsuta* ^d^	++	–	–	–					

^a^RG, rice-green manure rotation; RM, rice monoculture; RC, rice-crayfish co-culture; RD, rice-duck co-culture. +++, ++, and + represented occurrence of the listed weed species in 3, 2, and 1 sampling field/s, respectively; -, no occurrence of the listed weed species.

^b^Grass weeds.

^c^Sedge weeds.

^d^Broadleaf weeds.

**Figure 2:**
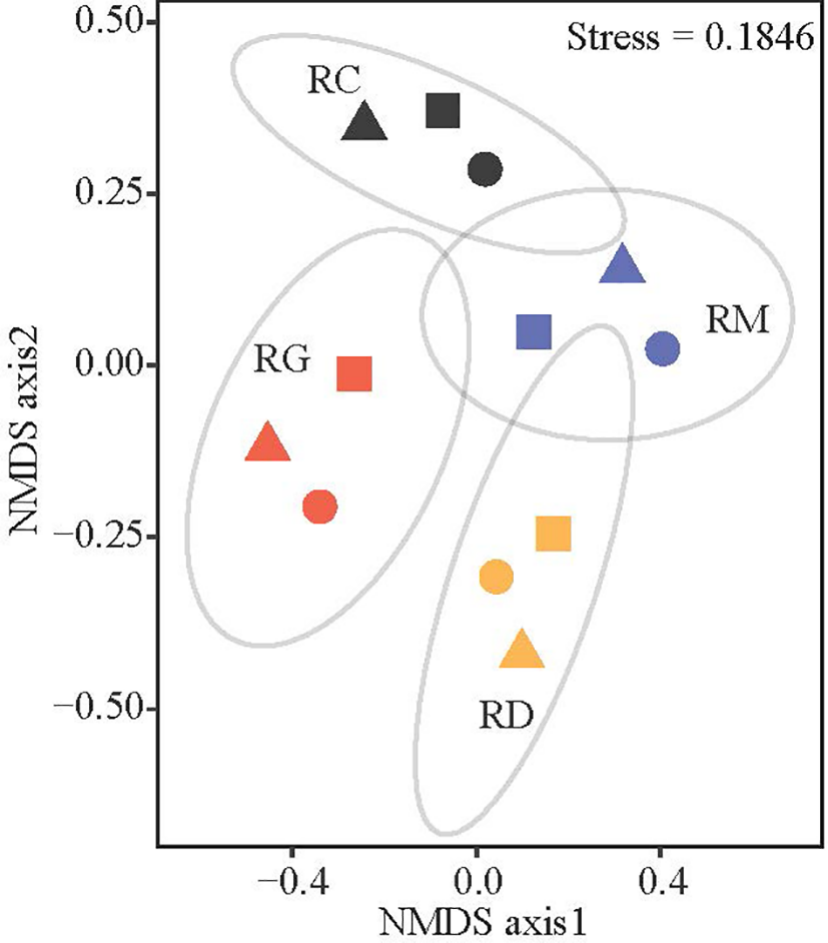
Nonmetric multidimensional scaling (NMDS) ordinations of weed communities based on their important value (*IV*) composition in the soil seed bank derived from Bray-Curtis distances matrices. In the figure, triangles, circles and squares represent weed communities in the fields in the north, middle and south of Jiangsu Province, respectively. Symbols with the color of red, blue, orange and black represent weed communities in the fields of rice-green manure rotation (RG), rice monoculture (RM), rice-duck co-culture (RD) and rice-crayfish co-culture (RC), respectively. Ellipses represent 95% confidence interval.

The NMDS results showed that the fields with the same organic production modes were slightly clustered, which indicated that the species composition in the soil weed seed bank varied according to organic production mode rather than sampling site (**Figure 2**). The ordinations of the 95% confidence intervals showed that the composition of the weed communities in the seed bank under RG, RC and RD were partly similar to that under RM but were different from each other. PERMANOVA is widely used to compare the significance of community structure differences among groups and to analyze how much of the total variance can be explained by grouping factors (Qian et al., 2020). The PERMANOVA results indicated that there were significant differences (*P* = 0.001) in community composition in the soil weed seed bank among different organic rice production modes, and the organic rice production mode explained 82% (*R* ^2^ = 0.82) of the total variation in weed community composition (data not shown).

**Figure 3:**
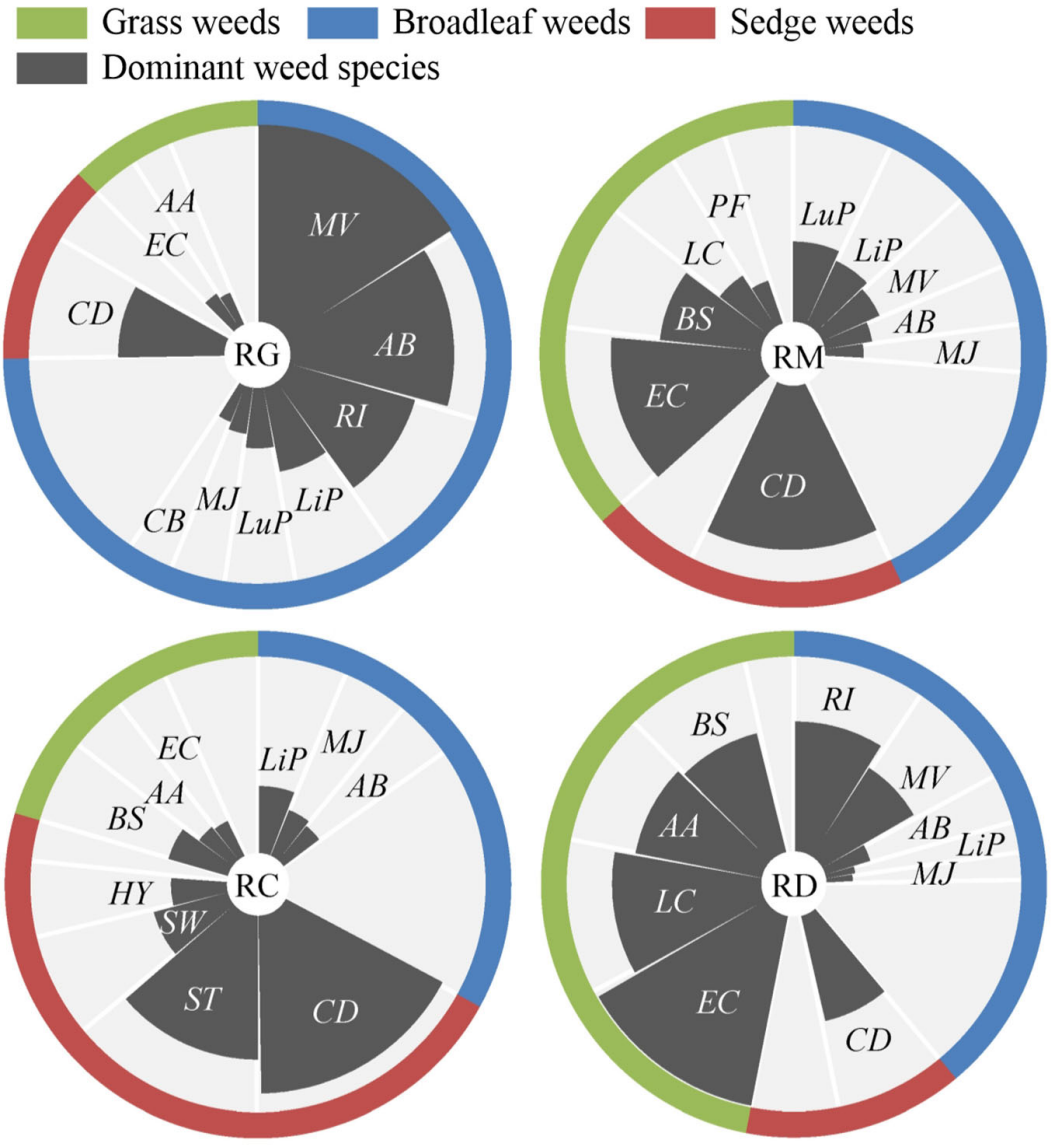
The composition of grass, broadleaf and sedge weeds and the ten dominant weed species in the soil seed bank (by important value of all three sampling fields) of different organic rice production modes. RG, rice-green manure rotation; RM, rice monoculture; RC, rice-crayfish co-culture; RD, rice-duck co-culture. *AA, Alopecurus aequalis*; *AB, Ammannia baccifera*; *BS, Beckmannia syzigachne*; *CB, Capsella bursa-pastoris*; *CD, Cyperus difformis*; *EC, Echinochloa crusgalli*; *HY, Heleocharis yokoscensis*; *LC, Leptochloa chinensis*; *LiP, Lindernia procumbens*; *LuP, Ludwigia prostrata*; *MJ, Mazus japonicus*; *MV, Monochoria vaginalis*; *PF, Polypogon fugax*; *RI, Rotala indica*; *ST, Scirpus triangulatus*; *SW, Scirpus wallichii*.

The *IV* values were used to determine the composition of grass, broadleaf and sedge weeds and the dominant weed species in the soil seed bank under different organic rice production modes (**Figure 3**). Broadleaf weeds were the dominant weed group, which accounted for 74.76% of the total *IV* value, followed by sedge (12.82%) and grass (12.42%) weeds, and *Monochoria vaginalis*, *A. baccifera*, *Rotala indica*, *C. difformis*, *L. procumbens*, *L. prostrata*, *M. japonicus*, *E. crusgalli*, *Capsella bursa-pastoris* and *A. aequalis* were the top ten dominant weed species in the seed bank under the RG mode. In the soil seed bank under the RM mode, broadleaf weeds were also the dominant weed group, accounting for 42.98% of the total *IV* value, but the sedge weed *C. difformis* and grass weeds *E. crusgalli* and *B. syzigachne* were the three most dominant weed species, followed by *L. prostrata*, *L. procumbens*, *M. vaginalis*, *L. chinensis*, *A. baccifera*, *Polypogon fugax* and *M. japonicus*. In the soil seed bank under the RC mode, sedge weeds were the most dominant weed group, which accounted for 46.51% of the total *IV* value, followed by broadleaf (33.04%) and grass (20.45%) weeds, and *C. difformis*, *Scirpus triangulatus*, *Scirpus wallichii*, *L. procumbens*, *B. syzigachne*, *Heleocharis yokoscensis*, *M. japonicus*, *A. baccifera*, *A. aequalis* and *E. crusgalli* were the top ten dominant weed species. Grass weeds were the dominant weed group, which accounted for 46.94% of the total *IV* value, followed by broadleaf (38.96%) and sedge (14.10%) weeds, and *E. crusgalli*, *L. chinensis*, *A. aequalis*, *R. indica*, *B. syzigachne*, *C. difformis*, *M. vaginalis*, *A. baccifera*, *L. procumbens* and *M. japonicus* were the top ten dominant weed species in the soil seed bank under the RD mode.

**Figure 4:**
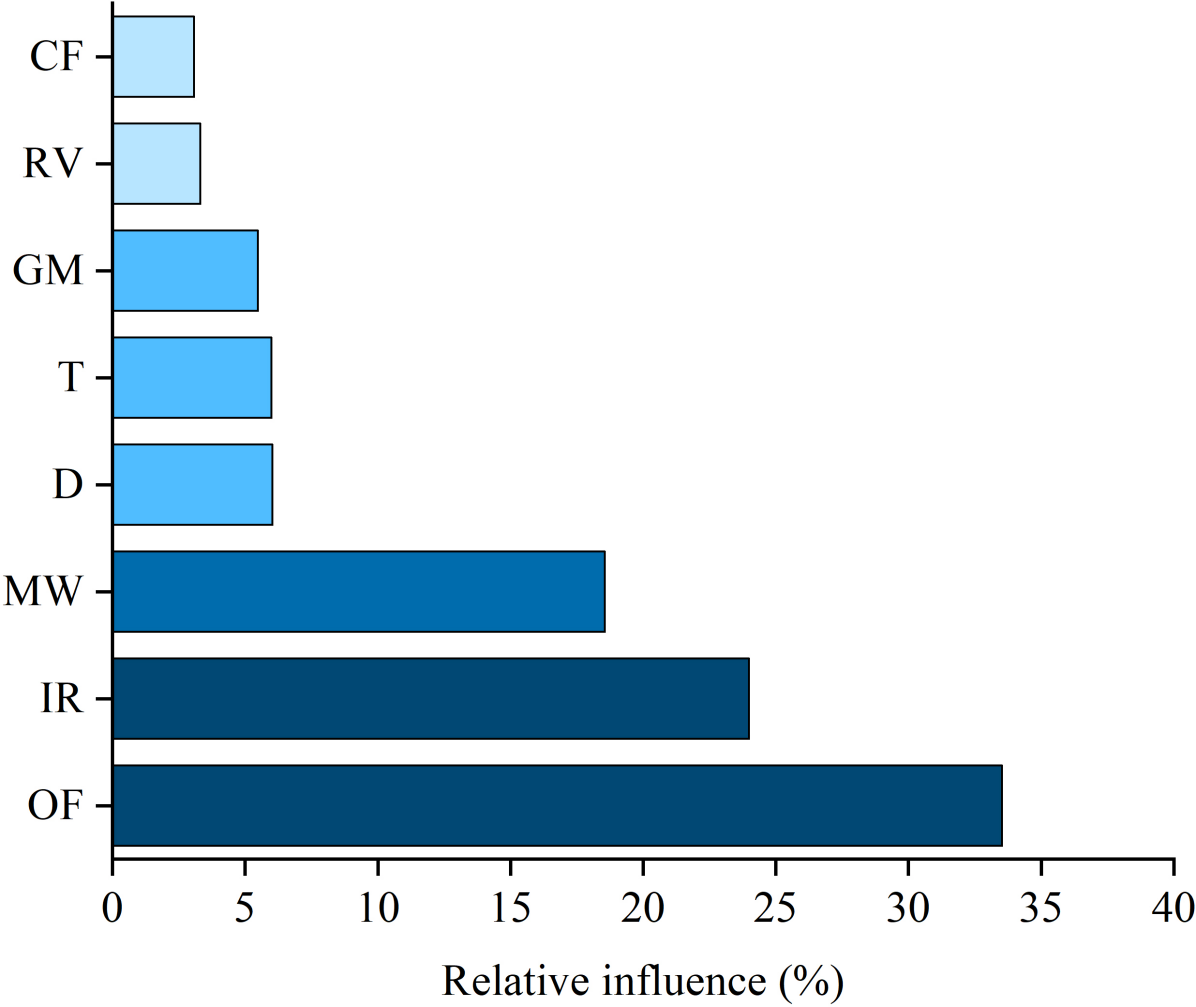
Aggregated boosted tree (ABT) analysis showed the relative influence of farming practices and frequency of hand weeding on the composition of weed community in the soil seed bank assigned to functional guild. OF, Annual application amount of organic fertilizer (kg·ha^-1^); IR, Annual irrigation volume (m^3^·ha^-1^); HW, Annual hand weeding time (hours·ha^-1^); D, Annual input of duckling (duckling·ha^-1^); GM, Fresh weight of green manure buried every year (kg·ha^-1^); T, Times of tillage per year; RV, Rice varieties; CF, Annual input of juvenile crayfish (kg·ha^-1^).

The ABT model is good at analyzing the nonlinearity and interaction between variables and quantitatively evaluating the relative influence of each explanatory variable on the response variables (De'ath, 2007). Therefore, ABT models were employed to interpret the relative influence of cultivation practices and frequency of hand weeding on the composition of the weed community in the soil seed bank (**Figure 4**). The annual application amount of organic fertilizer, irrigation volume and hand weeding frequency were identified as primary factors associated with the differences in the composition of weed communities, with relative influences of 33.53%, 24.00% and 18.56%, respectively.

**Figure 5:**
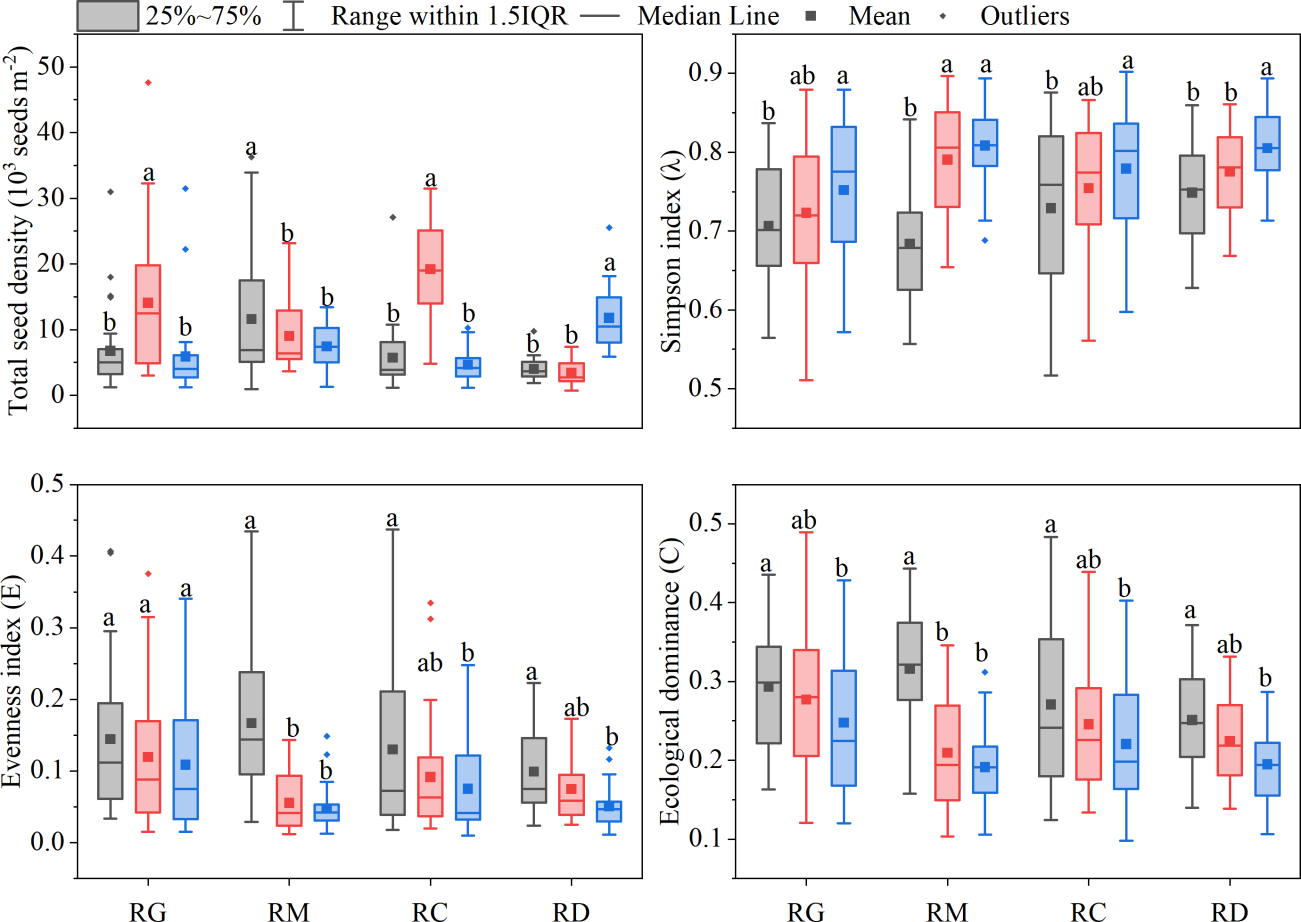
The variations of total seed density and diversity of weed communities in soil seed bank among consecutive planting years of different organic rice production modes. Gray box: consecutive planting for less 5 years; Red box: consecutive planting for more than or equal to 5 years but less than 8 years; Blue box: consecutive planting for more than or equal to 8 years. Boxes with the different lowercase letters are significantly different with different consecutive plantings years in the same organic production mode as determined by the LSD test at P< 0.05.

The variations in the total seed density and diversity of weed communities in the soil seed bank among consecutive planting years under the different organic rice production modes are presented in **Figure 5**. There were significant differences in the total seed density of the soil seed bank among consecutive planting years under all four organic production modes. However, these differences varied according to the number of consecutive planting years under the different organic production modes. The *λ* values of the weed community in the soil seed bank were significantly affected by the number of consecutive planting years and showed an increasing trend with the increase in consecutive planting years under all four organic production modes. Therefore, it indicated that the weed biodiversity increased with the increase in consecutive planting years under all four organic production modes. Although there was no significant effect of number of consecutive planting years on the *E* value of the weed community in the soil seed bank under the RG mode, the mean *E* value decreased with the increase in the number of consecutive planting years. In contrast, the *E* values of the weed communities in the soil seed bank under the RM, RC and RD modes were significantly affected by the number of consecutive planting years and decreased with the increase in consecutive planting years. Similarly, the *C* values of the weed community in the soil seed bank were significantly affected by the number of consecutive planting years and decreased with increasing number of consecutive planting years under all four organic production modes.

## Discussion

Weeds are plant species associated with crops that negatively affect crop growth and yield by competing with them for the same resources, mainly sunlight, water and nutrients (Smith et al., 2010; Keller et al., 2014). Therefore, changes in farming practices for crops, such as tillage (Chauhan and Opena, 2012), fertilization (Yin et al., 2005), irrigation (Mahajan et al., 2014), weeding methods and continuous and rotation cropping (Chamanabad et al., 2009; Santínmontanyá et al., 2013), affect the composition, density and diversity of weed communities in crop fields. As mentioned above, the RG, RM, RC, and RD modes are associated with a set of relatively independent farming practices for organic rice planting. To date, there are few reports on the composition of weed communities in organic rice fields, which were only partly analyzed in a few studies on rice-duck co-culture. A study on the influence of rice–duck farming on weed community composition for four consecutive years demonstrated that the control effect of rice-duck farming on broadleaf weeds was better than that on grass weeds, and the weed density in rice fields decreased with the increase in the number of consecutive years, among which the density of *E. crusgalli* decreased the most slowly (Wei et al., 2006). Zhao et al. (2014) reported that *A. aequalis* and *B. syzigachne* were always the most dominant weed species in wheat field seed banks over 13 consecutive years of rice-duck and wheat rotation farming. Similarly, our results showed that the grass weeds *E. crusgalli*, *A. aequalis* and *B. syzigachne* were the most dominant species in the soil seed bank under the RD mode. In contrast, the broadleaf weeds *M. vaginalis*, *A. baccifera* and *R. indica* and the sedge weeds *C. difformis*, *S. triangulatus* and *S. wallichii* were the most dominant species in the soil seed bank under the RG and RC modes, respectively. The soil seed bank of the RM mode was also dominated by broadleaf weeds, however, the most dominant weed species were sedge weed *C. difformis* and grass weeds *E. crusgalli* and *L. chinensis*. Therefore, more attention should be given to the control of grass weeds in the RD mode; while broadleaf weeds and sedge weeds should be the primary control targets in the RG and RC modes, respectively; besides, attention should also be paid to the outbreak of broadleaf weeds during the control of the dominant weeds *C. difformis*, *E. crusgalli* and *L. chinensis* in the RM mode; when formulating weed management strategies for organic rice fields.

Fertilizer is one of the major input costs in cropping systems, and its application is considered crucial for determining the competition relationship between crops and weeds, and it affects the species composition and biodiversity of weeds by stimulating plant growth and modulating competition for aboveground (e.g., light and space) and belowground (soil nutrients) resources (Yin et al., 2005; Zhang et al., 2019). The ABT analysis results indicated that the application amount of organic fertilizer made the largest contribution to the difference in weed composition of the soil seed bank under different organic rice production modes. The growth of green manure in the RG mode decreased the application amount of organic fertilizer, and in the RC and RD modes, the crayfish and duck waste and forage overapplication increased the nutrient supply for rice and reduce its dependence on organic fertilizers; therefore, the application amount of organic fertilizer and nutrient sources differed among the RG, RM, RD and RC modes. A study on the impacts of long-term composted manure and straw amendments on rice-associated weeds in a rice–wheat rotation system indicated that *A. baccifera*, *E. crusgalli*, and *M. vaginalis* could gain competitive advantages in fertile or infertile soil, while nutrient deficiency was more favorable to the infestation of sedge weeds (Gao et al., 2022). In our study, *A. baccifera*, *E. crusgalli*, and *M. vaginalis* were also dominant in the soil seed bank of all four organic rice production modes (except for *M. vaginalis* in the RC mode), and the lower input of organic fertilizer could be one of the reasons for the higher infestation of sedge weeds in the RC mode. Water is a powerful selective agent for weed management in paddy fields, and flooding is a key factor influencing the severity of weed competition (Kim et al., 2001; Caton et al., 2002). To provide a more suitable living environment for ducks and crayfish, the amount of irrigation in the RD and RC modes was higher than that in the RG and RM modes. Ducks and crayfish can prey on weed seedlings (Pirdashti et al., 2015; Yuan et al., 2021), which reduces the inputs required for hand weeding in the RD and RC modes. Our results indicated that although hand weeding and duck and crayfish breeding directly affected weed density, their influence on the variation in weed composition in the soil seed bank was lower than that of irrigation. Correspondingly, we also found that although the weed composition was similar under the same organic rice production mode at different sampling sites, there were significant differences in the total weed seed density in the soil seed bank. Wei et al. (2006) reported that the weed species richness and diversity decreased in an organic rice-duck coculture field over four years of consecutive planting. Our results indicated that weed species diversity increased and weed community evenness and dominance decreased with the increase in the number of consecutive planting years under all four organic rice production modes. The differences in the responses of weed species diversity in the RD mode may be due to the smaller number of consecutive planting years of rice-duck coculture in the study of Wei et al. (2006). Similarly, a nine-year study by Guo et al. (2020) demonstrated that weed diversity in the first four consecutive planting years of rice-crayfish coculture (with chemical fertilizer application but without weeding) showed a downward and then upward trend.

## Conclusion

Our results indicated the weed composition differed in the soil seed bank among the organic rice production modes of rice-green manure rotation, rice monoculture, rice-crayfish coculture, and rice-duck coculture. The most dominant weed group was broadleaf weeds in the soil seed bank of the rice-green manure rotation. The soil seed bank of the rice monoculture was also dominated by broadleaf weeds; however, the most dominant weed species were sedge and grass weeds. Sedge and grass weeds dominated the soil seed bank of rice-crayfish coculture and rice-duck coculture, respectively. Therefore, specific weed management strategies could be formulated based on the compositional differences in the weed community under different organic rice production modes. The application of organic fertilizer and irrigation had greater effects on the weed composition in the soil seed bank than hand weeding. Consequently, fertilization and irrigation strategies that alter weed composition could be used as improved weed management program components in organic rice production systems. Unlike the biodiversity loss in the long-term conventional rice planting which based on chemical fertilizer and herbicide, long-term organic rice planting was beneficial for increasing weed diversity in paddy fields. Our results also indicated that weed community evenness and dominance decreased with the increase in the number of consecutive planting years under all four organic rice production modes.

## Data availability statement

The raw data supporting the conclusions of this article will be made available by the authors, without undue reservation.

## Author contributions

QD contributed to the conception of the study and helped perform the analysis with constructive discussions; PG, HW, SD, and ED performed the field survey and contributed significantly to analysis and manuscript preparation; PG performed the data analyses and wrote the manuscript. All authors contributed to the article and approved the submitted version.

## Funding

This study was financially supported by the Key Research & Development Program of Jiangsu Province (BE2019343), the Postdoctoral Science Foundation of China (2020M671616), the Jiangsu Natural Science Foundation (BK20220565) and the National Key Research and Development Program of China (2021YFD1700805).

## Acknowledgments

We would like to thank Department of Agriculture and Rural Development of Jiangsu Province for help with the survey and data collection.

## Conflict of interest

Author ED was employed by Anhui Pujiwei Modern Agriculture Group Co., Ltd.

The remaining authors declare that the research was conducted in the absence of any commercial or financial relationships that could be construed as a potential conflict of interest.

## Publisher’s note

All claims expressed in this article are solely those of the authors and do not necessarily represent those of their affiliated organizations, or those of the publisher, the editors and the reviewers. Any product that may be evaluated in this article, or claim that may be made by its manufacturer, is not guaranteed or endorsed by the publisher.
